# Chrysin Modulates Genes Related to Inflammation, Tissue Remodeling, and Cell Proliferation in the Gastric Ulcer Healing

**DOI:** 10.3390/ijms21030760

**Published:** 2020-01-23

**Authors:** Felipe Leonardo Fagundes, Graziele de Morais Piffer, Larissa Lucena Périco, Vinicius Peixoto Rodrigues, Clélia Akiko Hiruma-Lima, Raquel de Cássia dos Santos

**Affiliations:** 1 Post Graduate Program in Health Sciences, São Francisco University, Bragança Paulista, SP CEP 129160-400, Brazil; felipel_fagundes@hotmail.com (F.L.F.); grazi.mpiffer@hotmail.com (G.d.M.P.); 2 Snyder Institute of Chronic Disease, Cumming School of Medicine, University of Calgary, Calgary, AB T2N 4N1, Canada; larissaperico@hotmail.com; 3 Department of Physiology, São Paulo State University “Júlio de Mesquita Filho”-UNESP, Biosciences Institute, Botucatu, SP CEP 18618-689, Brazil; viniciuspr42@gmail.com (V.P.R.); clelia.hiruma@unesp.br (C.A.H.-L.)

**Keywords:** chrysin, gastric ulcer, gastroprotective effect, tissue remodeling, healing

## Abstract

Chrysin exhibits anti-inflammatory and antioxidant activities. Here, the gastroprotective effect of chrysin was investigated in mouse models of gastric ulcer induced by absolute ethanol, acetic acid, and ischemia-reperfusion injury. The gastric-healing effect was evaluated at 7 and 14 days after treatment; the mechanism of action was verified using the expression of metalloproteinase 2 (*MMP-2*) and 9 (*MMP-9*), caspase-3, cyclooxygenase 1 (*COX-1*) and 2 (*COX-2*), epidermal growth factor (*EGF*), and interleukin-10. Chrysin (10 mg/kg) inhibited macroscopic lesions and increased catalase activity in the mouse model established using absolute ethanol. It ameliorated the gastric ulcer caused by acetic acid by improving the expression of inflammatory genes such as *COX-2*, inhibiting negative remodeling promoted by MMP-9, increasing cell proliferation effect via EGF, and reducing cellular apoptosis by modulating caspase-3. A faster healing effect was evident in the first 7 days of treatment compared to 14 days of treatment, indicating the pharmacological potential of chrysin. Overall, these results demonstrate the potent effect of chrysin in the gastrointestinal tract and elucidate the genes involved in the healing of gastric ulcers. Moreover, an increase in the levels of gastric mucosa defensive factors is involved in the activity of chrysin in the gastric mucosa.

## 1. Introduction

A peptic ulcer is a lesion in the gastric mucosa or duodenal lining; it is a condition that affects populations worldwide. The problem is further aggravated by the indiscriminate use of anti-inflammatory drugs and non-steroidal anti-inflammatory drugs (NSAIDs) for various purposes [1]. Gastrointestinal diseases tend to progressively increase with aging and thus an increased incidence is observed in aging populations [2]. The most frequent and severe complication of peptic ulcers is bleeding, which occurs in 1 in 1000 individuals each year and continues to be one of the most common causes of hospitalization [3]. The bleeding is often caused by the presence of the bacterium *Helicobacter pylori* and/or the use of NSAIDs [4]. Determining the etiology of peptic ulcers is highly difficult due to the heterogeneity of this disease [5,6]. It is considered a disease of modern times, accelerated by factors such as a busy lifestyle (stress), common vices (tobacco and alcohol), and an unbalanced diet, and it is often fatal [7,8]. The available treatments for this disease include the use of proton pump inhibitors (PPIs), histamine receptor antagonists, and antibiotics. However, these treatments result in the development of several adverse effects [1], such as gastroenteric reactions, rebound effects, hypergastrinemia, hepatic and renal toxicities, and gastric adenocarcinoma [2,3,4,5]. Alternative treatments using compounds isolated from some plants have been developed to alleviate the severity of ulcerative diseases. Flavonoids are a group of polyphenolic compounds derived from plants, and they can be categorized as simple phenols, phenolic acids, coumarins, condensed and hydrolysable tannins, lignans, and lignins [6]. Chrysin is a flavone found naturally in honey, propolis, and plant species such as *Passiflora caerulea* L. [7]. Its antioxidant, anti-inflammatory, anti-colorectal, neuroprotective, hepatoprotective, cardioprotective, and antidiabetic activities have been reported, which suggest the therapeutic potential of chrysin [8,9,10,11]. Therefore, in this study, we evaluated the potential gastroprotective effect and healing action of chrysin in different experimental mice models and assessed the potential underlying mechanisms of action. 

## 2. Results and Discussion

**Figure 1:**
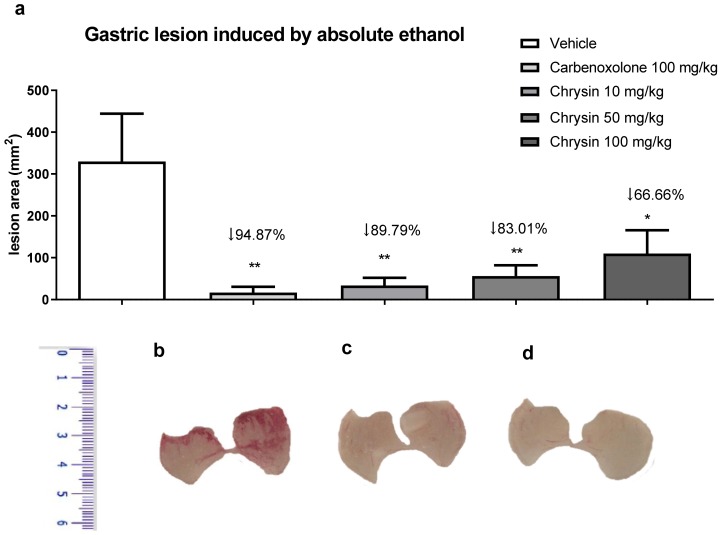
(**a**) Effect of chrysin in the model of gastric ulcer induced by absolute ethanol. The bar represents the mean ± S.E.M. of lesion area, and statistical significance was determined using the ANOVA followed by Dunnett’s test; * *p* < 0.05 and ** *p* < 0.01 compared with the vehicle. Macroscopic photographs of the stomachs of mice treated with (**b**) vehicle 10 mL/kg, (**c**) carbenoxolone 100 mg/kg, and (**d**) chrysin 10 mg/kg.

Alcoholism, besides smoking, stress, and *H. pylori* infection, is considered a risk factor for peptic ulcers. The chronic consumption of ethanol leads to gastric ulceration, reducing mucus production, cell proliferation, and gastric blood flow and promoting an inflammatory response [12]. Figure 1 shows the protective effect of chrysin in the model of gastric ulcer induced by absolute ethanol. We observed that chrysin at the lower dose of 10 mg/kg reduced 89.79% of the lesions (Figure 1), whereas at doses of 50 and 100 mg/kg, it reduced the lesions by 83.01% and 66.66%, respectively, compared with that of the vehicle-treated group (*p* < 0.01). Chrysin, at a dose ten times smaller, similar to the positive control, carbenoxolone, inhibited the occurrence of gastric lesions promoted by absolute ethanol at all tested doses. The lowest effective dose of chrysin, 10 mg/kg, was used in the subsequent experiments because there was no significant difference among the tested doses.

Ethanol can easily penetrate the gastric mucosa, and the lesions observed in the gastric mucosa can be attributed to the production of reactive oxygen species, rupture of endogenous mucus, secretion of gastric acid [13], damage to the gastric mucosa related to hemorrhagic lesions, apoptosis, induction of lipid peroxidation and oxidative stress, and reduction in reduced glutathione (GSH) and prostaglandin levels [14]. The cellular damage increases neutrophil infiltration in the gastric tissue, resulting in the generation of free radicals that consequently aggravate the lesions [15].

**Figure 2:**
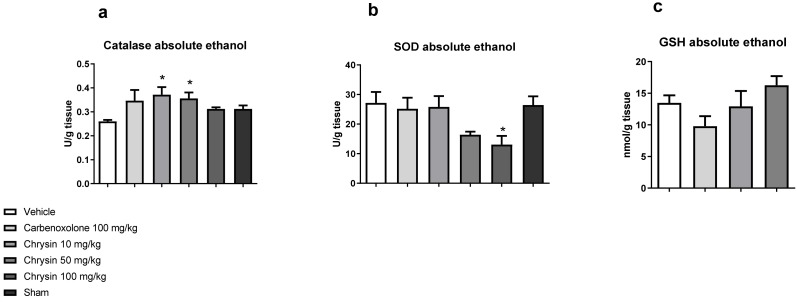
Effect of chrysin on the redox system: (**a**) catalase, (**b**) superoxide dismutase, and (**c**) reduced glutathione in the gastric mucosa of mice with gastric ulcer induced by absolute ethanol. The bar represents mean ± S.E.M., and statistical significance was determined using the ANOVA followed by Dunnett’s test; * *p* < 0.05 compared with the vehicle.

After determining the protective effect of chrysin, the next step was to elucidate the underlying mechanism of action. Figure 2 shows the effects of chrysin on the main antioxidant enzymes involved in the protection of gastric mucosa. Chrysin elevated the catalase activity at doses of 10 (43.1%) and 50 mg/kg (37.1%) and reduced the superoxide dismutase (SOD) activity at the dose of 100 mg/kg (51.96%) compared with those of the vehicle-treated group but did not affect the GSH level (*p* > 0.05). 

The cytoprotective effect of chrysin treatment was characterized by the improvement in the activity of catalase, the most important enzyme involved in free radical scavenging. The carbonyl group at C-4 and double ligation at C-2 and C-3 are strongly responsible for the antioxidant activity of chrysin [16]. 

**Figure 3:**
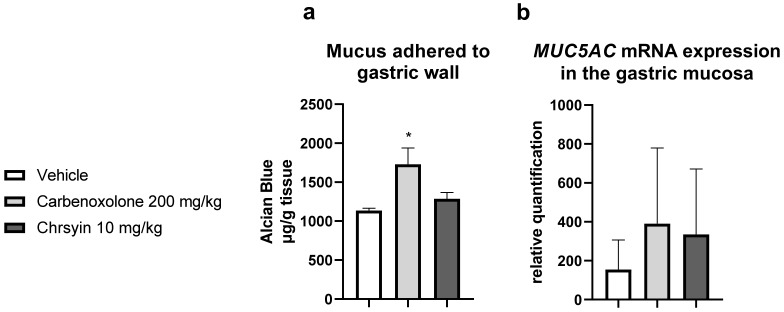
Effects of chrysin on (**a**) the production of adherent gastric mucus (measured as the amount of bound Alcian blue) and (**b**) relative expression of *MUC5AC* in the gastric mucosa of mice with ulcer induced by absolute ethanol. The bar represents mean ± S.E.M., and statistical significance was determined using the ANOVA followed by Dunnett’s test; * *p* < 0.05 compared with the vehicle.

Mucus is one of the components of the “gastric mucosal barrier,” which covers the mucosal surface [17]. In the present study, chrysin did not change the level of mucus adhering to the gastric wall compared with that in the vehicle group. Furthermore, it did not change the expression of *MUC5AC*, an important gene related to the production of mucus in the stomach (Figure 3).

**Figure 4:**
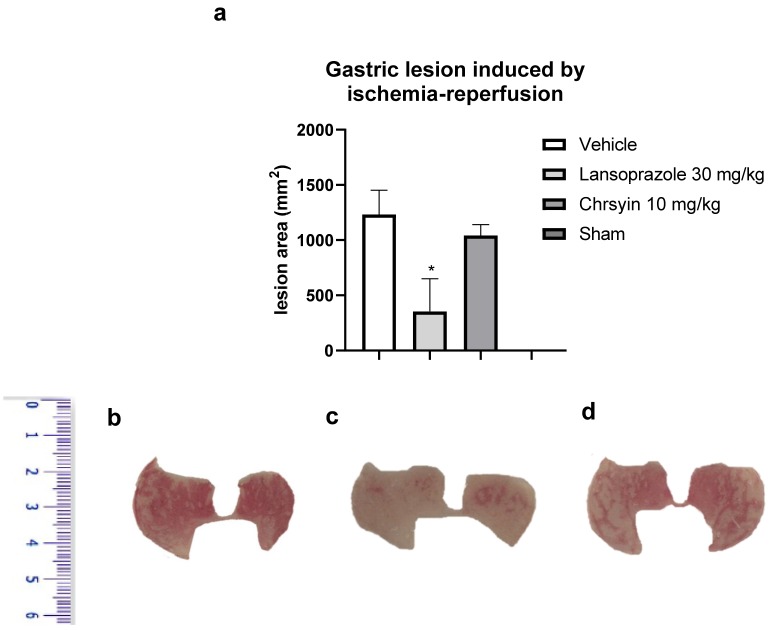
(**a**) Effect of chrysin in the model of gastric ulcer induced by ischemia-reperfusion. The bar represents the mean ± S.E.M. of lesion area, and statistical significance was determined using the ANOVA followed by Dunnett’s test; * *p* < 0.05 compared with the vehicle. Macroscopic photographs of the stomachs of mice treated with (**b**) vehicle 10 mL/kg, (**c**) lansoprazole 30 mg/kg, and (**d**) chrysin 10 mg/kg.

Ischemia-reperfusion has deleterious effects on the gastric mucosa and is one among several stress-induced gastric mucosal injuries [17]. In this study, we developed a mouse model of gastric ulcer induced by ischemia-reperfusion. Gastric ulcer lesions are caused by alterations in blood flow (ischemia) and anaerobic metabolism of the tissue. The reintroduction (reperfusion) of blood into the tissue aggravates the lesions caused by ischemia with the release of proinflammatory substances and generation of reactive oxygen species [18]. These molecules lead to an elevation in neutrophil infiltration, producing more free radicals and inflammatory mediators [19,20]. In the present study, in the model of ischemia-reperfusion, chrysin (10 mg/kg) did not protect the gastric mucosa, compared to the vehicle-treated group (Figure 4). On the contrary, lansoprazole (30 mg/kg), a proton pump inhibitor, reduced the macroscopic lesion area, compared to the vehicle, and protected the gastric mucosa (*p* < 0.05; Figure 4). These data agree with those obtained with GSH, another antioxidant factor that was not altered by the action of chrysin. 

One of the least understood aspects of ulcers is the chronicity of the disease, which is characterized by repeated episodes of healing and exacerbation, posing a challenge to physicians and a burden for patients [21]. The acetic-acid-induced gastrointestinal ulcer model is a classical model that has been proven to be suitable for investigating the effect of treatment on the healing process of chronic gastrointestinal ulcers and search for new anti-ulcer drugs [22]. Such data can also help identify new anti-ulcer drugs or treatment targets. It remains unclear whether chrysin has any beneficial effects in the healing of induced gastric ulcers, as the gastroprotective effect of a compound does not ensure its gastric-healing effect in induced gastric ulcers [23]. 

**Figure 5:**
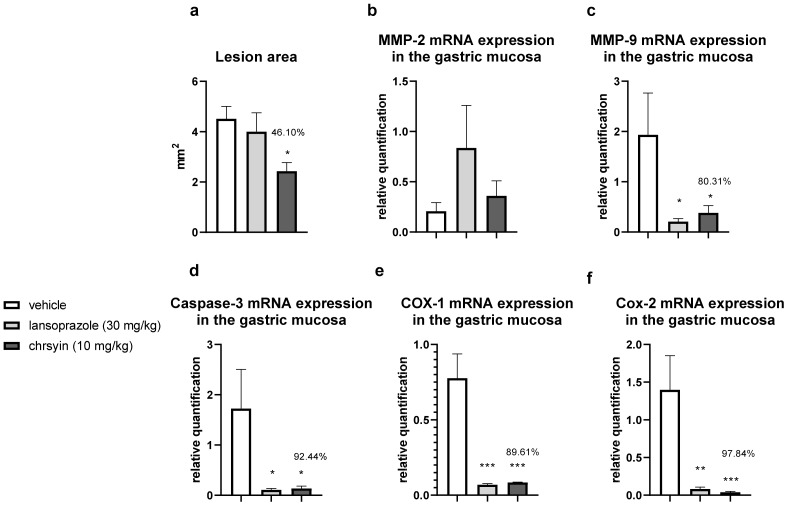
Effect of chrysin in the model of gastric ulcer induced by acetic acid. (**a**) Macroscopic lesion area and relative quantification of mRNA expression of (**b**) *MMP-2*, (**c**) *MMP-9*, (**d**) *caspase-3*, (**e**) *COX-1*, and (**f**) *COX-2* after 7 days of treatment. The bar represents the mean ± S.E.M. of lesion area, and statistical significance was determined using the ANOVA followed by Dunnett’s test; * *p* < 0.05, ** *p* < 0.01, *** *p* < 0.001 compared with the vehicle.

**Figure 6:**
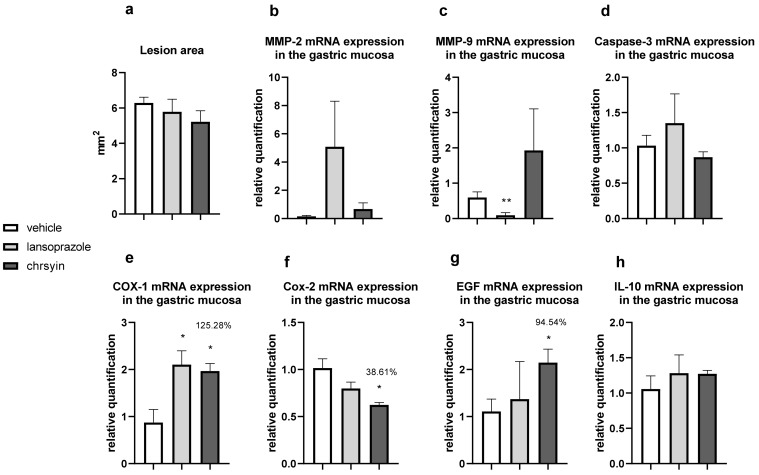
Effect of chrysin in the model of gastric ulcer induced by acetic acid. (**a**) Macroscopic lesion area and relative quantification of mRNA expression of (**b**) *MMP-2*, (**c**) *MMP-9*, (**d**) *caspase-3*, (**e**) *COX-1*, (**f**) *COX-2*, (**g**) *EGF*, and (**h**) *IL-10* after 14 days of treatment. The bar represents the mean ± S.E.M. of lesion area, and statistical significance was determined using the ANOVA followed by Dunnett’s test; * *p* < 0.05 and ** *p* < 0.01 compared with the vehicle.

Figure 5 shows the healing activity of chrysin after 7 days of treatment in the model of gastric ulcer induced by acetic acid. Chrysin reduced the macroscopic lesion area by 46.1% compared with that in the vehicle-treated group (*p* < 0.05). However, with 14 days of treatment (Figure 6), chrysin did not reduce gastric lesions significantly compared with those in the vehicle-treated group (*p* > 0.05). 

The healing process of gastric ulcers can be divided into the following phases: ulcer-development phase (0–3 days), involving tissue necrosis, ulcer implantation, inflammatory infiltration, and ulcer margin formation; rapid-healing phase (3–10 days), involving migration of epithelial cells and contraction of the ulcer base; and slow-healing phase (10–20 days), involving angiogenesis, granulation tissue remodeling, and complete ulcer re-epithelialization [24]. To understand the molecular role of chrysin in the healing process, we investigated several genes implicated in the healing process. After 7 days of treatment, chrysin reduced the expression of MMP-9, caspase-3, COX-1, and COX-2 compared with that in the vehicle-treated group (*p* < 0.05, Figure 6). The administration of chrysin for 14 days did not reduce the gastric lesion area but improved cell proliferation promoted by EGF and the inflammatory status influenced by COX-2. In addition, chrysin elevated the relative expression of COX-1 but did not significantly alter the expression of MMP-2 and -9 (*p* > 0.05, Figure 6). 

The matrix metalloproteinases (MMPs) are a part of the endopeptidase group that degrades the extracellular matrix, which is fundamental in the process of remodeling and healing. MMP-2 and MMP-9 are gelatinases that regulate the main pathways of cellular signaling responsible for growth, migration, inflammation, and angiogenesis [25]. Some studies have shown that an imbalance in MMPs leads to a deficiency in angiogenesis and poor healing of gastroduodenal ulcers [26]. Other studies have reported that the ulceration process induced by acetic acid involves an elevation in the gelatinases, which return to normal levels during the healing process [27]. Li et al. [28] demonstrated that an increase in MMP-9 in gastric tissue is often associated with the development and progression of gastric ulcers. In the present study, we observed a reduction in MMP-9 in the chrysin-treated group, compared with that in the vehicle-treated group (Figure 6), which is consistent with that demonstrated in myocardial infarction [29]. Chrysin did not increase the expression of MMP-2, an important regulator of angiogenesis, compared with that in the vehicle-treated group (Figure 6). After 14 days of treatment, we observed the same effects on the expression of MMP-2 and MMP-9 (Figure 6). 

Chrysin is involved, at least partially, in the reduction of apoptosis mediated by caspase-3, an effector of cell death that breaks the nuclear components responsible for cytoskeleton formation [30]. This effect was observed only after treatment for 7 days (*p* < 0.05, Figure 6). Next, we evaluated the expression of cyclooxygenase; this enzyme has two isoforms, COX-1 (constitutive), which is involved in gastric protection, and COX-2 (inducible), which is elevated in the inflammatory process. Chrysin reduced the expression of COX-1 and COX-2 after 7 days of treatment (Figure 6) but elevated the expression of COX-1 and reduced the expression of COX-2 after 14 days of treatment (Figure 6). A possible explanation is that, after 2 weeks, the tissue recovered and, therefore, there was a reduction in the inflammatory status. 

Reconstruction of epithelial surface is essential for gastric ulcer healing and it is controlled by factors such as EGF, a factor released by the epidermal cells that inhibits acid secretion, exerts a trophic effect in the gastric mucosa, and mediates mucosal adaptation. EGF is implicated in the growth and development as well as the protection of the gastrointestinal tract, besides repairing the gastric mucosa after injury [31,32]. Chrysin did not elevate the expression of EGF after 7 days of treatment (Figure 6); however, after 14 days of treatment, it increased the level of EGF in the treated group compared with that in the vehicle-treated group (Figure 6). This suggested that chrysin may act to induce cell proliferation in the model of gastric ulcer induced by acetic acid, a key process in the gastroduodenal healing. 

Another important event associated with gastric healing is inflammation. The results of the present study showed that chrysin did not significantly increase the expression of IL-10 (Figure 6) in both treatment groups. IL-10 is a cytokine produced by activated macrophages that downregulate inflammation and release some inflammatory mediators in the inflamed tissue. It plays a critical role in the depletion of inflammatory cytokines, which results in the downregulation of inflammation [33]. Chrysin demonstrated anti-inflammatory and angiogenic potential. It can act in the rapid-healing phase and slow-healing phase, wherein angiogenesis, tissue remodeling, and ulcer re-epithelialization play critical roles [24]. Despite the promising results presented so far, the use of natural products in humans should be undertaken cautiously, particularly when the optimal dose and duration of therapy have not been established. However, chrysin displays promising activities in the low doses studied, justifying further investigation.

## 3. Materials and Methods

### 3.1. Animals

The in vivo experimental procedures were conducted using male Swiss mice (*Mus musculus*) obtained from Multidisciplinary Center for Biological Investigation in the Area of Laboratory Animals (Unicamp-SP-Brazil); they were divided into the following groups (5–6 individuals/group): negative control (saline solution, 0.9% NaCl), positive control (lansoprazole 30 mg/kg or carbenoxolone 100 mg/kg), chrysin (10, 50 and 100 mg/kg-Cayman, Ann Arbor, MI, USA) and sham (exposed to the stress of fasting and procedure/surgery). The animals were fed Presence^®^ diet, with water ad libitum and maintained under controlled conditions of illumination (12:12 light/dark cycle) and temperature (22 ± 2 °C). All animals were fasted (10 h) before the assays. The mice were kept in cages of polypropylene. All the experimental practices were approved by the Ethics Committee in Experimental Animal Care of the São Francisco University (protocol 001.06.2016 date 26 August 2016).

### 3.2. Evaluation of Gastric Protective and Healing Effects of Chrysin

#### 3.2.1. Gastric Lesions Induced Absolute Ethanol [34]

Male mice (*n* = 5) were separated in the following groups and deprived of food for 12 h, with water ad libitum. The mice of different groups received the following treatments: vehicle (saline 10 mL/kg), carbenoxolone (100 mg/kg), and chrysin (10, 50, and 100 mg/kg, Sigma Aldrich). The absolute ethanol at an oral dose of 0.2 mL/animal was administered 60 min after the pretreatment. After 60 min of ethanol administration, the animals were euthanized, and the stomachs were collected for analysis of gastric lesions using the software AvSoft BioView Spectra (São Paulo, Brazil). All treatments were given by oral gavage. 

#### 3.2.2. Quantification of Catalase Activity [35]

To determine the activity of catalase, fragments of gastric mucosa were homogenized in RIPA buffer with protease inhibitor cocktail. After homogenization, the samples were centrifuged at 13,680 × *g* for 15 min at 4 °C. The supernatant was diluted in the following buffer: KH_2_PO_4_ (phosphate dihydrogen potassium) 25 mM pH 7.5, EDTA 1 mM, BSA 1% in the ratio of 1:20 (*w*/*v*). Tissue homogenate (20 µL) was placed in 96-well plate with 100 µL of assay buffer (KH_2_PO_4_ 250 mM pH 7.0), 30 µL of methanol, and 20 µL of hydrogen peroxide (35.3 mM). The plate was incubated for 10 min at room temperature. After that, 15 µL of potassium periodate (65.2 mM) was added to the wells, and the plates were again incubated at room temperature. The absorbance was read at 540 nm and the final concentrations were multiplied by 4000. Results were expressed in U/g tissue. All experiments were conducted in duplicate.

#### 3.2.3. Quantification of Superoxide Dismutase Activity [36]

Strips of the stomach were homogenized in RIPA buffer with protease inhibitor cocktail. After homogenization, the samples were centrifuged in 13,680× *g* for 15 min at 4 °C. The supernatant was diluted in phosphate buffer (0.1 M pH 7.4) in the proportion of 1:20 (*w*/*v*). To 100 µL of tissue homogenate was added 150 µL of cocktail containing: hypoxanthine, xanthine oxidase and nitro blue tetrazolium (NBT) in the ratio 1:1:1. The absorbance was read each minute during a period of ten minutes, at 37 °C and 560 nm. The integral was multiplied by 800, and the results were expressed in U/g tissue. All experiments were conducted in duplicate.

#### 3.2.4. Quantification of Reduced Glutathione Levels [37]

Fragments of gastric mucosa were homogenized in RIPA buffer with protease inhibitor cocktail. After homogenization, the samples were centrifuged in 13,680× *g* for 15 min at 4 °C. In a 96-well plate, 100 µL of samples (diluted in 0.1 M PB, pH 7.4, 1:10 *w*/*v*) and 100 µL Tris/EDTA buffer were added and read at 412 nm. After the first read, 20 µL of 5,5′-dithio-bis-2-nitrobenzoic acid (DTNB) was added and the plate was read again at 412 nm for 15 min. The concentration was multiplied by 10 and the results were expressed in nmol/g of tissue. All experiments were conducted in duplicates

#### 3.2.5. Quantification of Mucus Adhered to Gastric Wall [38]

Confirmation that chrysin elevates the mucus production was realized by the quantification of adhered mucus according to [38]. Briefly, animals (*n* = 6) were fed for 8 h and pretreated with vehicle (saline 10 mL/kg), positive control (carbenoxolone 200 mg/kg) and chrysin (10 mg/kg). All treatments were given by oral gavage. After, one hour of treatment, mice received absolute ethanol in the dose of 0.2 mL/animal. After another hour, the animals were euthanized and the glandular area of the stomach were placed in a solution of Alcian blue (Alcian blue *w*/*v* 0.1%, sucrose, 0.16 mol/L, sodium acetate 0.05 mol/L, pH 5.8) for two hours. The stomachs were washed in 0.25 M sucrose solution and kept in 0.5 M magnesium chloride for 2 h while shaking them periodically. After two hours had elapsed from the last procedure, the stomachs were removed, dried, weighed, and discarded. Diethyl ether was added to the solution in the ratio 1:1; after shaking and centrifugation (3874× *g*) the supernatant was discarded, and the concentration of Alcian blue was measured in a spectrophotometer with λ = 598 nm. The results are expressed as µg of Alcian blue/g of tissue.

#### 3.2.6. Gastric Ulcer Induced by Ischemia-Reperfusion [39]

Mice (*n* = 6) were divided in different groups: negative control (saline 10 mL/kg), positive control (lansoprazole 30 mg/kg), and test group (chrysin 10 mg/kg). The treatments were given by oral gavage. One hour before the surgical procedure, the animals received the treatments (vehicle, lansoprazole, and chrysin). After 1 h, the animals were anesthetized with xylazine and ketamine (0.4 and 0.8 mg/kg, respectively, intramuscular) and had their abdomens opened to exposure of celiac artery. This vessel was isolated, and a microvascular clamp was placed, blocking the blood flow for 30 min. After this period, the clamp was removed (reperfusion), and 60 min were allowed to elapse before the animals were euthanized for the macroscopic analysis of lesion.

#### 3.2.7. Gastric Ulcer Induced by Acetic Acid [40,41,42]

Male Swiss mice (*n* = 6) were kept in fasting with water ad libitum for 8 h before the experiment. After anesthesia, a laparotomy with an epigastric incision was made in all animals. A plastic micro tube of 4.2 mm diameter was pressed under the serosa wall, where 35 µL of acetic acid 80% *v*/*v* was applied for 20 s in the serosae surface and completely removed from the stomach using a saline solution. After the procedure, the abdomen was sutured, and the animals were fed normally. Chrysin (10 mg/kg), lansoprazole (30 mg/kg), or vehicle (saline 10 mL/kg) were given daily for 7 or 14 days starting one day after the surgery. One day after the last administration of treatments, the animals were euthanized, and the stomachs were collected for analysis of lesions. All treatments were given by oral gavage.

#### 3.2.8. Gastric Mucosa RNA Extraction and mRNA Studies [43,44]

The total RNA extraction from stomach biopsies was proceed according to a protocol utilizing trizol (Sigma Aldrich). Briefly, the samples were homogenized with 300 µL of trizol reagent, and 200 µL of chloroform was added to tissue homogenate, following incubation in 4 °C for 10 min. After that, samples were centrifuged in 12,000× *g* at 4 °C for 10 min, and the aqueous phase was precipitated with a mixture of 20 µL of sodium acetate 3 M, 1 µL of glycogen (10 µg/µL, Gen Elute LPA-Sigma Aldrich), and 500 µL of isopropanol, before a new incubation for 10 min at 4 °C. The samples were centrifuged, and the supernatant was discarded. Subsequently, 500 µL of alcohol 75% (*v*/*v*) was added to the pellet and after new centrifugation, 12,000× *g* at 4 °C for 10 min, the solvent was withdrawn. After complete evaporation, the pellets were resuspended in an adequate volume of nuclease free water (Sigma Aldrich, St Louis, MO, USA) and the concentration of total RNA was measured by spectrophotometry using the equipment Nanodrop 2000 (Thermo Scientific, Wilmington,, DE, USA). The qPCR was developed using QuantiTect SYBR Green I (Qiagen, Hilden, Germany) according to the manufacturer recommendations. The experiments were made in duplicates of four distinct samples and the results were normalized with the expression levels of a housekeeping gene (β-actin). Expression of MMP-2, MMP-9, caspase-3, COX-1, COX-2, EGF, IL-10 and β-actin was determined using the specific primers. MMP-2 was determined using the 5′-GGACAGTGACACCACGTGAC-3′ forward and 5′-TGACACAGCCTTCTCCTCCT-3′ reverse primers (GenBank 17390). MMP-9 was determined using 5′-CGTCGTGATCCCCACTTACT-3′ forward and 5′-AACACACAGGGTTTGCCTTC-3′ reverse primers (GenBank 17395). Caspase-3 was determined using 5′-GGCCGTGTTTCTGTTTTGTT-3′ forward and 5′-TTGAGGTAGCTGCATGTGG-3′ reverse primers (GenBank 12367). COX-1 was determined using 5′-AGGGTGTCTGTGTCCGCTTT-3′ forward and 5′-GTTGGGGACTGGAGTCTTGC-3’ reverse primers (GenBank 19224). COX-2 was determined using 5′-CCCCAAAGATAGCATCTGGA-3′ forward and 5′-TGCAGAATTGAAAGCCCTCT-3′ reverse primers (GenBank 19225). EGF was determined using 5′-AGGCATCAAGCACGGTAGGT-3′ forward and 5′-AGCAAGCACACCCCGTAAGT-3′ reverse primers (GenBank 13645). IL-10 was determined using 5′-AAAAGGTGCCACCCTGAAGA-3′ forward and 5′-GATGTGGTGGGGACCAACCTT-3′ reverse primers (GenBank 16153). β-actin was determined using 5′-ACGAGGCCCAGAGCAAGAG-3′ forward and 5′-GGTGTGGTGCCAGATCTTCTC’-3′ reverse primers (GenBank 11461). Primer pairs of these genes were designed based in the entire FASTA sequence using NCBI data base and using the Primer3 according to the following parameters: primer length of 20 bases, melting temperature around 60 °C, and amplicon size between 95–110 bases.

### 3.3. Statistical Analysis 

Results were expressed as mean ± standard error mean of the parameters obtained. The values obtained were subjected to one-way analysis of variance (ANOVA), followed by Dunnett’s test to compare three or more groups. Data were analyzed using GraphPad Prism 8.0 (San Diego, CA, USA) software. The results were considered significant when *p* < 0.05. 

## 4. Conclusions

The present study revealed, for the first time, the gastric-ulcer-healing and preventive actions promoted by chrysin against highly damaging agents and elucidated the molecular mechanism underlying its healing effect. These actions are modulated by increasing the catalase activity and modulating inflammation, tissue remodeling, and cell proliferation in gastric mucosa. These results demonstrated a significant potential of chrysin in alleviating gastric ulcers and elucidated the molecular mechanism underlying its healing effect. Moreover, they support further investigation of therapeutic use of this flavone in gastrointestinal disorders.

## Abbreviations

CAT	catalase
COX-1	cyclooxygenase 1
COX-2	cyclooxygenase 2
EGF	epidermal growth factor
GSH	reduced glutathione
IL-10	interleukin 10
MMP-2	metalloproteinase 2
MMP-9	metalloproteinase 9
MUC5AC	mucin 5ac
NSAID	non-steroidal anti-inflammatory drug
PPI	proton pump inhibitor
SOD	super oxide dismutase
